# Prostaglandin E1 Preconditioning Attenuates Liver Ischemia Reperfusion Injury in a Rat Model of Extrahepatic Cholestasis

**DOI:** 10.1155/2018/3812424

**Published:** 2018-01-08

**Authors:** Feng Xu, Xiaolin Liu, Chao Wang, Chaoliu Dai

**Affiliations:** Department of Hepatobiliary and Spleen Surgery, Shengjing Hospital Affiliated to China Medical University, Shenyang, Liaoning 110004, China

## Abstract

The aim of this study is to explore the hepatoprotective effect of intraportal prostaglandin E1 (PGE1) on liver ischemia reperfusion (IR) injury using an extrahepatic cholestatic model, observing oxidative stress markers, proinflammatory factors, apoptotic marker proteins, and an adhesion molecule. The extrahepatic cholestatic model was induced by common bile duct ligation. After seven days, rats were subjected to ischemia by Pringle maneuver for 15 min, followed by 1, 6, or 24 h of reperfusion. Prostaglandin E1 (PGE group) or normal saline (NS group) was continuously infused from 15 min before liver ischemia to 1 h after reperfusion. After reperfusion, histopathological evaluation of the liver was performed, as were measurements of bilirubin, biochemical enzymes, oxidative stress markers (GSH and MDA), proinflammatory factors (MPO, TNF-*α*, and IL-1*β*), apoptotic marker proteins (Bcl-2 and Bax), and the adhesion molecule (ICAM-1). PGE1 pretreatment attenuated IR injury in extrahepatic cholestatic liver probably by suppressing MDA, MPO, TNF-*α*, IL-1*β*, ICAM-1, and Bax levels and improving GSH and Bcl-2 levels. In conclusion, PGE1 protects extrahepatic cholestatic liver from IR injury by improving hepatic microcirculation and reducing oxidative stress damage, intrahepatic neutrophil infiltration, and hepatocyte apoptosis.

## 1. Introduction

Many patients with extrahepatic cholestasis, such as hilar cholangiocarcinoma, need to undergo liver resection for radical resection of lesions, which is an effective means of long-term survival [1, 2]. In order to reduce postoperative complications and mortality, it is extremely important to minimize bleeding during liver parenchymal dissection. Pringle maneuver, one type of hepatic inflow occlusion, has been widely used to control hemorrhage during liver resection but induces liver ischemia reperfusion (IR) injury.

Cholestasis can damage sinusoidal endothelial cells, induce hepatic microcirculation dysfunction, and render the liver susceptible to warm IR compared to normal liver [3–5]. IR will further impair microvascular perfusion defects in extrahepatic cholestatic livers rather than only unrestrained oxidative/nitrosative stress [5]. Preoperative biliary drainage has been shown to reduce hepatic IR after ischemia in the animal study [4]. However, there is still controversy in clinic about the risks and benefits of preoperative biliary drainage [6–8].

There are several approaches to prevent or attenuate liver IR injury, like ischemic preconditioning, pharmaceutical pretreatment, antioxidant preconditioning, gene targeting, and others [9]. Drug pretreatment has been proved to be an effective means of protection against liver IR injury. Prostaglandin E1 (PGE1), which can improve the liver microcirculation by the expansion of blood vessels and antiplatelet aggregation and increase its energy metabolism and bile secretion, can protect liver IR injury [10]. PGE1 can also downregulate expression of adhesion and inflammatory modulating molecules and reduce hepatocytic degeneration [11]. Furthermore, short oxygenated warm perfusion combined with PGE1 could reduce hepatocyte necrosis and apoptosis and ameliorate mitochondrial permeability transition in liver grafts [12]. Although the previous study had reported that a low dose of PGE1 infusion could improve hepatic arterial and portal venous blood flow in the extrahepatic cholestatic model [13], its mechanism against IR injury in extrahepatic cholestatic liver has not been elucidated. The aim of this study is to evaluate the hepatoprotective effect of intraportal PGE1 on liver IR injury in a common bile duct ligated rat model.

## 2. Materials and Methods

### 2.1. Experimental Animals and Experimental Design

Male albino Wistar rats (SPF), weighing 250 to 300 g, were purchased from Central Animal House of Shengjing Hospital, an affiliate of China Medical University. They were housed in individual cages with wood-chip bedding and acclimatized for 7 days before any experimental procedures under standardized laboratory conditions in a temperature-controlled room with 12-hour dark/light cycles. Standard rodent chow and water were provided ad libitum to all the animals. This project followed our institution's criteria for care and use of laboratory animals in research, which conformed to the National Institutes of Health Guidelines.

Animals underwent bile duct ligation for 7 days and were randomly divided into two groups: the normal saline (NS, *n* = 18) control group and the prostaglandin E1 treatment (PGE, *n* = 18) group. The PGE group was infused a PGE1 solution in a dose of 0.5 *μ*g·kg^−1^·min^−1^ (purchased from Beijing Saisheng Pharmaceutical Co.) dissolved in normal saline at a rate of 2 ml/h using a portable infusion pump. The NS group received normal saline infused at the same speed. Each of the two groups was further evenly divided into three subgroups according to different reperfusion times.

### 2.2. Surgical Procedures

All surgical procedures were performed under a sterile environment. Rats were anesthetized with an intraperitoneal injection of 10% chloral hydrate. The model of extrahepatic bile duct obstruction and hepatic ischemia reperfusion was the same as previously reported [14]. A midline incision was performed on day 0, and the common bile duct (CBD) was ligated and transected. After 7 days, the rats underwent liver ischemia reperfusion and pharmacological pretreatment. Hepatic inflow was occluded by Pringle maneuver with an atraumatic clip for 15 min, and then the liver was reperfused by removing the clamp. Biliary reconstruction was made by cannulating the common bile duct to the duodenum with an epidural catheter. Pharmacological pretreatment was performed, such that the PGE group was continuously pretreated with intraportal injection of PGE1 solution for 15 min before liver ischemia and until the end of liver reperfusion for 60 min. The NS group was preconditioned with normal saline like PGE group.

The animals were sacrificed after 1, 6, and 24 h of reperfusion, and blood and liver samples were collected. Serum was separated and stored at −20°C until the levels of biochemical enzymes and bilirubin were measured. Liver tissues were frozen quickly and stored in liquid nitrogen for biochemical assays or fixed in 10% formalin for histological analysis.

### 2.3. Serum Detection of Biochemical Enzymes and Bilirubin

Total bilirubin (TBil), direct bilirubin (DBil), alkaline phosphatase (ALP), alanine aminotransferase (ALT), aspartate aminotransferase (AST), and lactate dehydrogenase (LDH) were measured using standard clinical methods with an autobiochemical analyzer (Hitachi, Japan).

### 2.4. Light Microscopic Examination

Liver tissue specimens of the left lateral lobe were biopsied and fixed in 10% (V/V) buffered formalin and embedded in paraffin. Sections were cut at 4 *μ*m intervals and stained with hematoxylin/eosin for histological examination with light microscopy. Liver damage was analyzed by two pathologists blindly using Suzuki's criteria [15].

### 2.5. Total Glutathione (GSH) and Malondialdehyde (MDA) Assay

Liver tissue samples were homogenized in ice-cold phosphate buffer (pH 7.4). The homogenates were centrifuged at 16,000*g* and 3,500*g* for 10 min at 4°C, and the supernatants were used for determination of total GSH levels and MDA content, respectively. Levels of GSH and MDA were assayed with commercial assay kits, which were purchased from the manufacturer (Nanjing Jiancheng Bioengineering Institute, Nanjing, China). The absorbance of the samples was assessed with a spectrophotometer at the wavelength of 412 nm and 532 nm, respectively.

### 2.6. Myeloperoxidase (MPO) Activity Assay

Liver MPO activity was determined in the liver tissue homogenates by methods suggested by the manufacturer and measured using a spectrophotometer at 460 nm. All reagents were purchased from Nanjing Jiancheng Biology Engineering Institute. MPO activity was expressed as units per gram of tissue.

### 2.7. Enzyme-Linked Immunosorbent Assay (ELISA)

Tissue samples were subsequently homogenized in ice-cold phosphate buffer (pH 7.4). The homogenates were centrifuged at 16,000*g* for 10 min at 4°C, and the supernatants were used for determining the levels of tumor necrosis factor-alpha (TNF-*α*) and interleukin-1 beta (IL-1*β*). Levels of TNF-*α* and IL-1*β* were assayed with commercial ELISA assay kits as indicated by the manufacturer (Senxiong Biotech Co. Ltd., Shanghai, China). The absorbance was assayed with a spectrophotometer at the wavelength of 492 nm.

### 2.8. Immunohistochemistry Assay

The expressions of intercellular adhesion molecule-1 (ICAM-1), Bcl-2, and Bax on paraffin-embedded liver tissue sections were examined by using SP method, as previously reported [14]. Five fields were randomly selected using an OLYMPUS-B5X microscope and scanned by the image analyzer system. Positive controls were used to set parameters, and all samples were measured under the same conditions. The averages of integrated optical density of ICAM-1, Bcl-2, and Bax (monoclonal rabbit anti-mouse primary antibodies, Santa Cruz, CA, USA) expressions were analyzed by the image analyzer software.

### 2.9. Statistical Analysis

All data were expressed as mean ± standard deviation (SD) and analyzed by Prism software (GraphPad 6). Two-way ANOVA with Sidak's multiple comparisons test was used to evaluate statistical significance. Values of *P* < 0.05 were considered statistically significant.

## 3. Results

### 3.1. Liver Function

**Figure 1:**
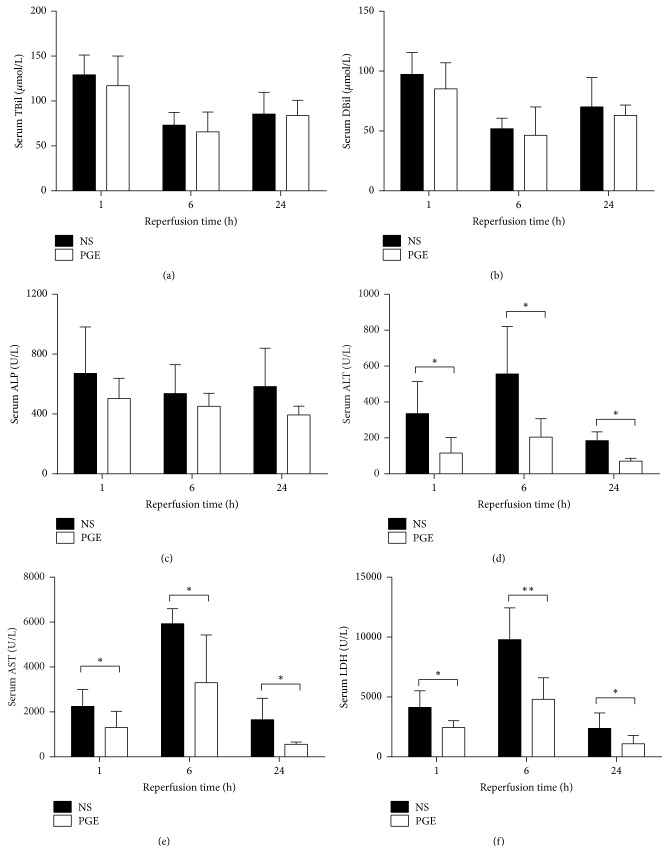
The serum levels of bilirubin (*μ*mol/L) and biochemical enzymes (ALP, ALT, AST, and LDH) (U/L) in PGE group and NS group at 1, 6, and 24 h after reperfusion. The data are expressed as the mean ± SD (*n* = 6, ^*∗*^*P* < 0.05, and ^*∗∗*^*P* < 0.001).

In the present study, the serum levels of TBil, DBil, and ALP in the PGE group were decreased at 1, 6, and 24 h, respectively, compared with those in NS group but had no statistical differences (*P* > 0.05, Figures 1(a)–1(c)). The serum levels of ALT, AST, and LDH reached their peak at 24 h after reperfusion and were significantly lower in the PGE group than those in the NS group at 1, 6, and 24 h of reperfusion, respectively (*P* < 0.05, Figures 1(d)–1(f)).

### 3.2. Histopathological Damage Degree of Liver

**Figure 2:**
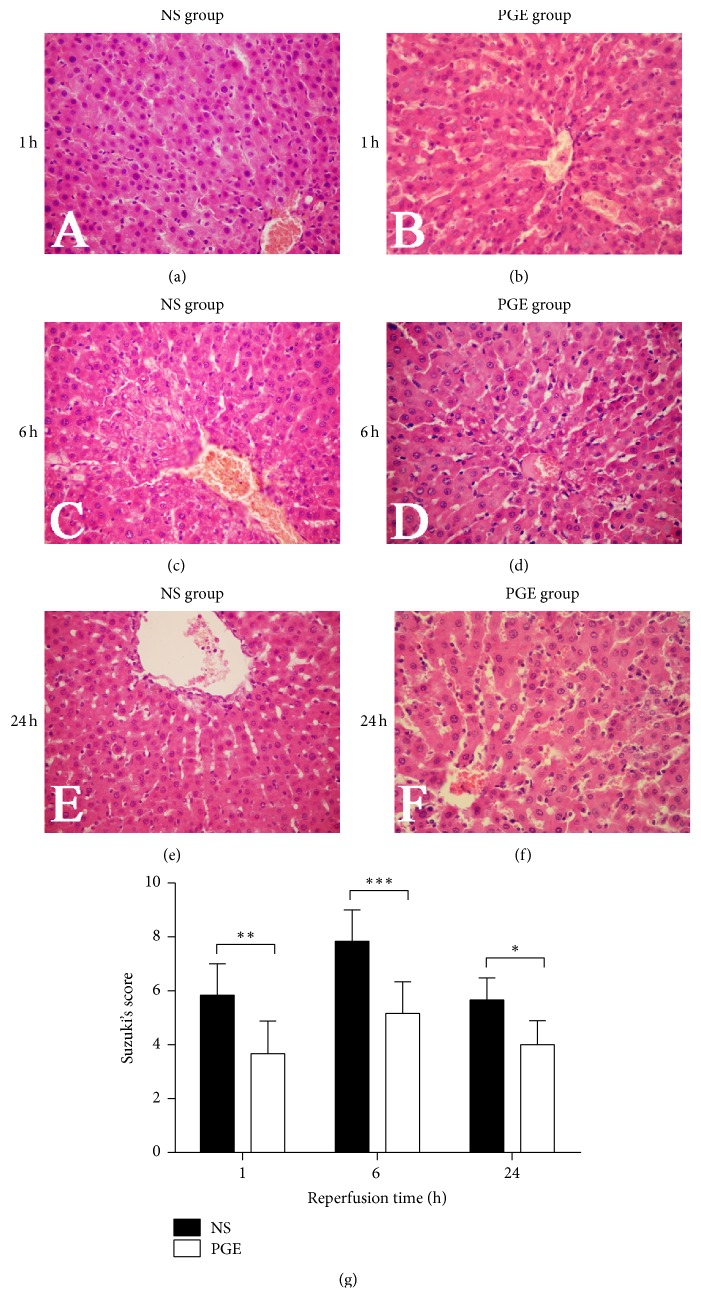
Histopathological changes in the liver tissue of each group at 1, 6, and 24 h after reperfusion. Representative hematoxylin-and-eosin (HE) stained microphotographs were taken from the NS group (a, c, and e) and the PGE group (b, d, and f) (original magnification, ×400). (g) Suzuki's score: the data are expressed as the mean ± SD (*n* = 6, ^*∗*^*P* < 0.05, ^*∗∗*^*P* < 0.001, and ^*∗∗∗*^*P* < 0.0001).

In the current study, histopathologic evaluation of cholestatic livers after IR injury displayed that hepatic cell became swollen, hepatic lobules were distorted, and the liver sinus became narrow and congested with neutrophils at 1 h after reperfusion in the NS group. At 6 h after reperfusion, the hepatic tissue structure became more disordered with massive necrosis area surrounding the central vein. At 24 h after reperfusion, the swelling of the hepatocytes slightly reduced and the liver sinus gap widened slightly. In contrast, the PGE group displayed slighter liver tissue damage than in the NS group. The observed changes include the following: decreased hepatic cell swelling; more regular hepatic cord arrangement; clearer and more dilated hepatic sinus; and significantly decreased necrosis areas after 6 h reperfusion (Figure 2).

### 3.3. Expression of GSH and MDA in the Liver Tissue

**Figure 3:**
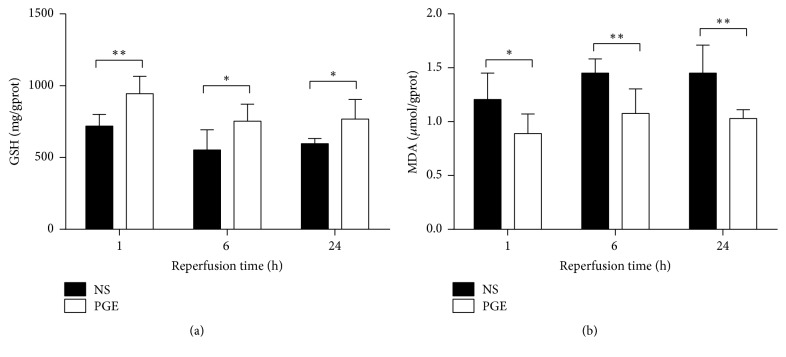
The levels of GSH and MDA in liver tissue in PGE group and NS group at 1, 6, and 24 h after reperfusion. The data are expressed as the mean ± SD (*n* = 6, ^*∗*^*P* < 0.05, and ^*∗∗*^*P* < 0.001).

In this experiment, the results showed that the levels of GSH were higher in the PGE group than those of the NS group, respectively, at 1, 6, and 24 h after reperfusion. The levels of MDA were significantly lower in the PGE group than those of the NS group, accordingly. The differences were statistically significant (*P* < 0.05) (Figure 3).

### 3.4. Expression of ICAM-1 in the Liver Tissue

**Figure 4:**
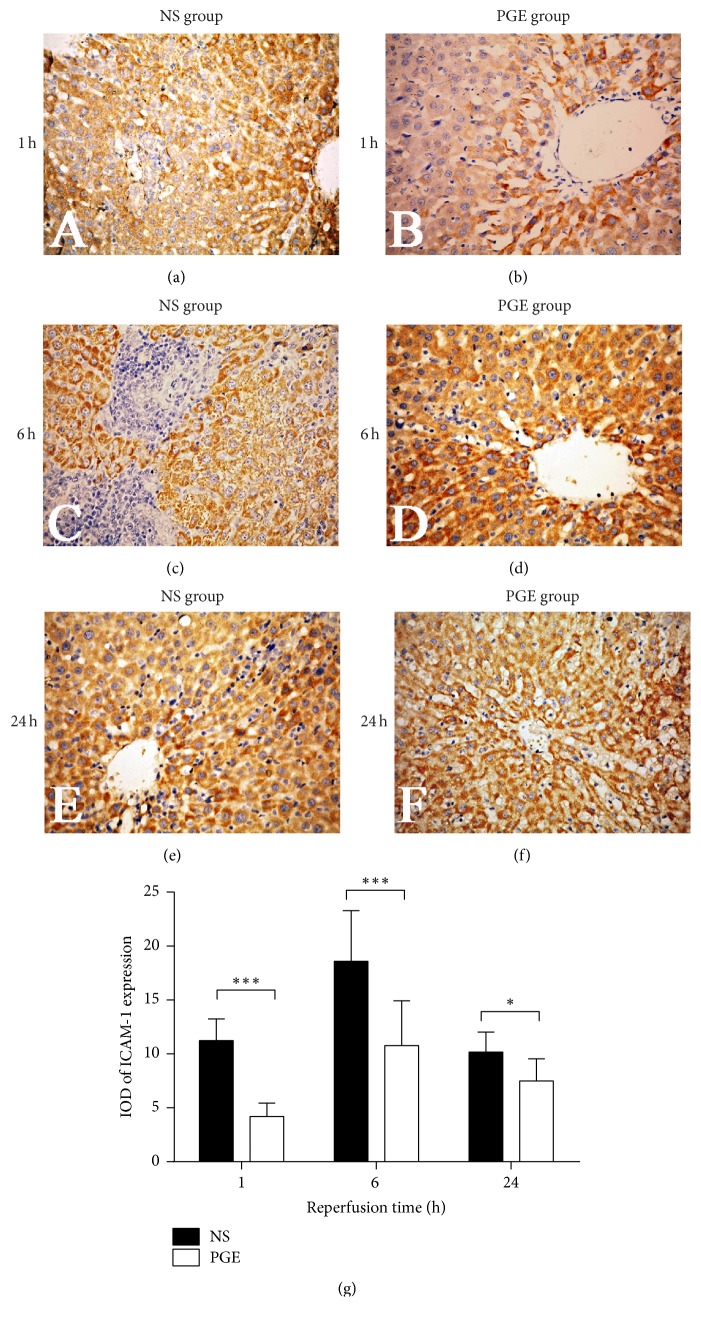
The expression of ICAM-1 in liver tissue of PGE group and NS group at 1, 6, and 24 h after reperfusion as measured by immunohistochemical staining (a–f, magnification, ×400). The average integral optical density (IOD) of ICAM-1 expression in the two groups: the data are expressed as the mean ± SD (*n* = 6, ^*∗*^*P* < 0.05, and ^*∗∗∗*^*P* < 0.0001).

The results showed that when compared with the NS group, PGE1 significantly lowered the levels of ICAM-1, at 1, 6, and 24 h after reperfusion, respectively. For both groups, ICAM-1 expression reached the peak at 6 h after reperfusion (Figure 4).

### 3.5. Expression of MPO and Proinflammatory Cytokines in the Liver Tissue

**Figure 5:**
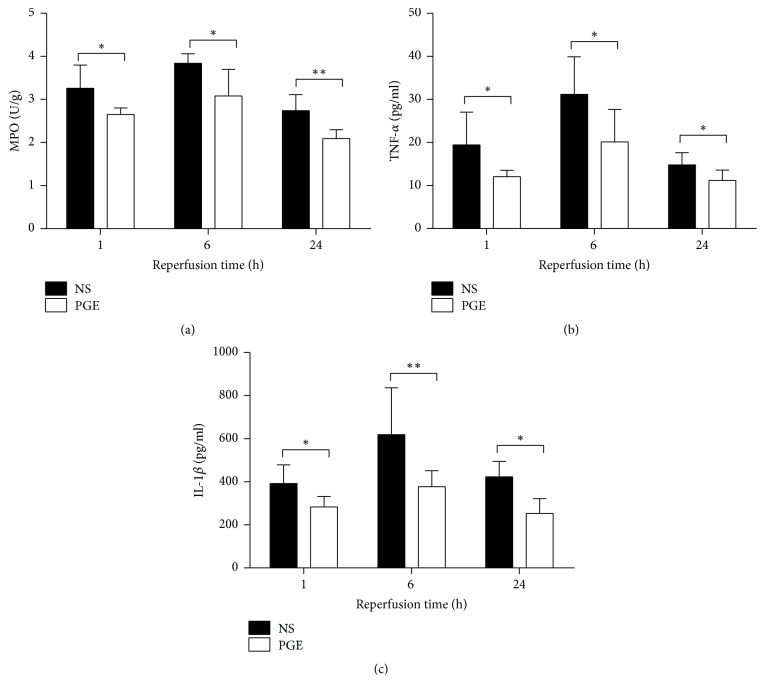
The levels of MPO, IL-1*β*, and TNF-*α* in liver tissue in PGE group and NS group at 1, 6, and 24 h after reperfusion. The data are expressed as the mean ± SD (*n* = 6, ^*∗*^*P* < 0.05, and ^*∗∗*^*P* < 0.001).

The results demonstrated that PGE1 pretreatment significantly reduced the levels of MPO, IL-1*β*, and TNF-*α* in liver tissue at 1, 6, and 24 h after reperfusion, respectively, when compared to the NS group. The levels of MPO, IL-1*β*, and TNF-*α* reached a peak at 6 h after reperfusion and then decreased in both of the two groups (Figure 5).

### 3.6. Expression of Bcl-2 and Bax in the Liver Tissue

**Figure 6:**
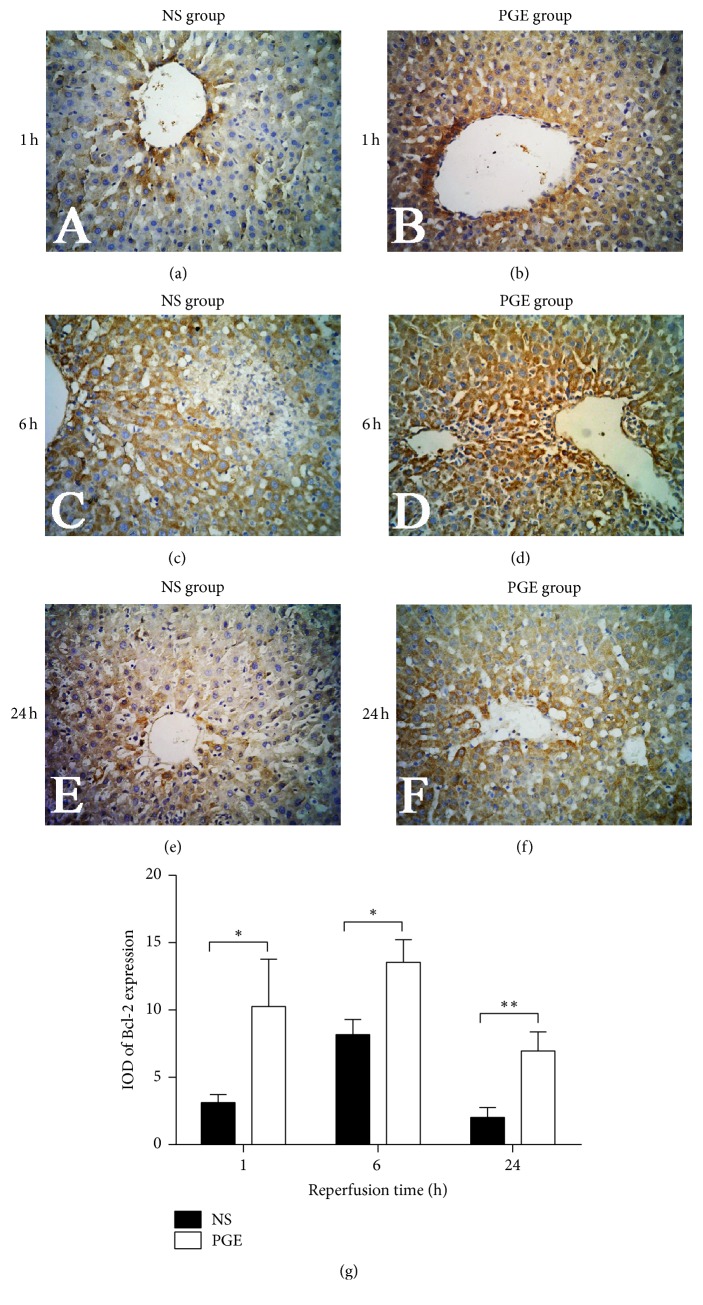
The expressions of Bcl-2 in liver tissue of PGE group and NS group at 1, 6, and 24 h after reperfusion as measured by immunohistochemical staining (a–f, magnification, ×400). The average integral optical density (IOD) of Bcl-2 expression in two groups: the data are expressed as the mean ± SD (*n* = 6, ^*∗*^*P* < 0.05, and ^*∗∗*^*P* < 0.001).

**Figure 7:**
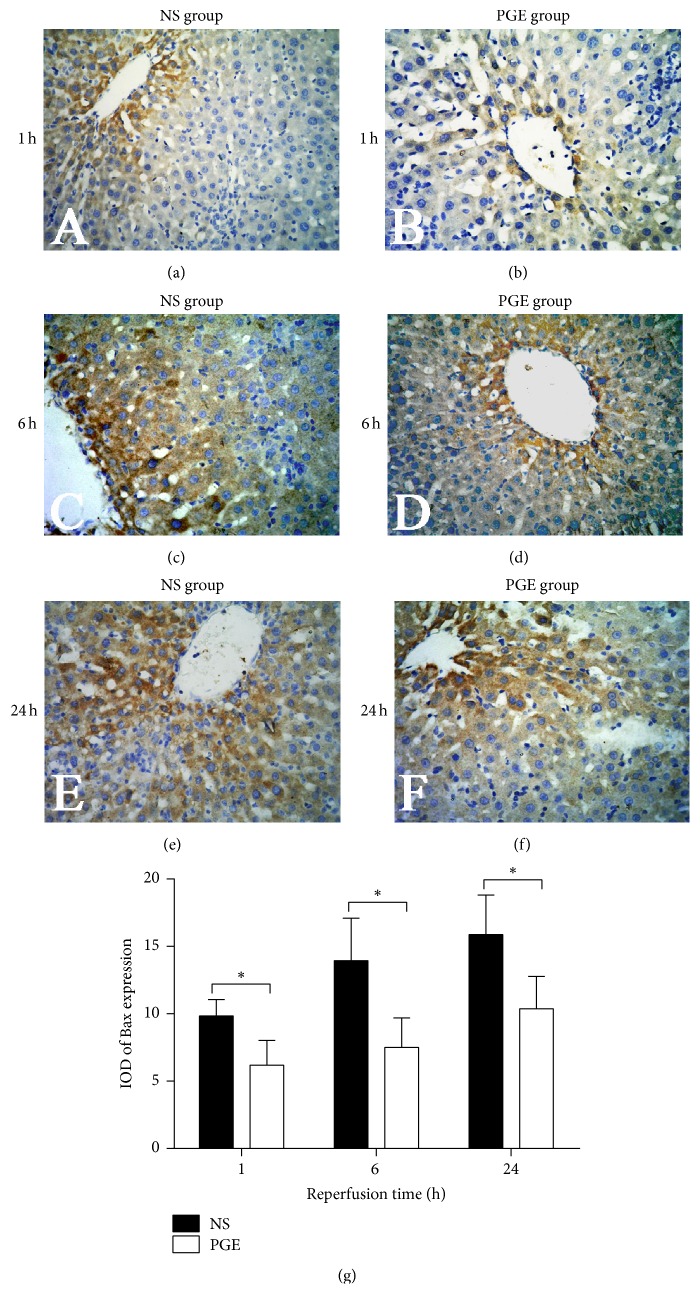
The expression of Bax in liver tissues of the PGE and NS groups at 1, 6, and 24 h after reperfusion as measured by immunohistochemical staining (a–f, magnification, ×400). (g) The average integral optical density (IOD) of Bax expression in two groups: the data are expressed as the mean ± SD (*n* = 6, ^*∗*^*P* < 0.05).

The results showed that PGE1 significantly enhanced the expression level of Bcl-2 in the PGE group versus the NS group. Furthermore, the level of Bax was significantly lower in the PGE group than in the NS group at 1, 6, and 24 h after reperfusion, respectively (Figures 6 and 7).

## 4. Discussions

This study aimed to investigate whether PGE1 pretreatment could relieve liver IR injury in cholestatic livers and the molecular mechanisms induced by PGE1. Here, our data showed that PGE1 preconditioning relieved liver IR injury by reducing oxidative stress, proinflammatory cytokines release, ICAM-1 expression, and hepatocyte apoptosis.

In this study, we found that PGE1 pretreatment significantly reduced the levels of serum ALT, AST, and LDH and ameliorated liver tissue damage. It is well known that ALT is localized exclusively from the hepatocellular cytoplasm, while both AST and LDH are liver mitochondrial and cytosolic enzymes [16, 17]. These enzymes are common indicators to evaluate the degree of hepatocyte damage [18]. ALT level was positively correlated with the degree and volume of liver cell necrosis in liver IR injury, and the correlation was more apparent at 24 h after reperfusion [19]. This study showed PGE1 pretreatment could reduce the release of enzymes, necrosis of hepatocytes, and apoptosis, which suggests a reduction in hepatocellular damage. We also further confirmed that these enzymes were correlated with the degree of liver cell necrosis. Furthermore, the intraportal infusion of PGE1 could improve hepatic arterial and portal venous blood flow [13]. PGE1 also could inhibit platelet aggregation to improve microcirculatory failure and hypoxia [20]. In this study, pathological damage of liver was characterized by dilation of hepatic sinusoids and reduced infiltration of neutrophils.

During reperfusion, reactive oxygen species is one of the major contributing factors to the onset of liver injury by activation of neutrophils and lipid peroxidation of membranes, which also is associated with a decrease in the antioxidant defense [21]. GSH is an endogenous antioxidant enzyme which could indirectly reflect the level of oxygen free radical and oxidative damage. MDA is a product of lipid peroxidation and also considered as an indirect index reflecting the state of oxidative damage [22]. In the present study, PGE1 preconditioning reduced GSH consumption and the generation of MDA. The results indicated that PGE1 improved microcirculation, leading to the effective oxygenation of liver tissue. This was consistent with previous studies. IR further impaired microvascular perfusion defects in extrahepatic cholestatic livers rather than only unrestrained oxidative/nitrosative stress [5]. PGE1 improved antioxidant capacity in liver tissue and effectively reduced hepatic IR injury [23].

MPO is a sensitive indicator of infiltrating neutrophils. TNF-*α* and IL-1*β* are two important cytokines which are released by the infiltrated neutrophils [24, 25]. Our study demonstrated that hepatic sinus gathered a large number of neutrophils especially at 6 h after reperfusion, and PGE1 pretreatment significantly decreased the levels of proinflammatory factors (MPO, TNF-*α*, and IL-1*β*). These results show that PGE1 reduces neutrophil infiltration and is consistent with previous studies [11, 20]. PGE1 inhibited the release of inflammatory cytokines, like TNF-*α*, probably mediated through the inhibition of activated Kupffer cells and of generated ROS [26]. This current study also showed PGE1 suppressed the expression of ICAM-1, in part by decreasing the release of TNF-*α*. TNF-*α* upregulates ICAM-1 expression, which aids neutrophils to roll, adhere, and migrate during parenchymal infiltration [27]. PGE1 may also inhibit hepatic sinus endothelial cells secretion of vascular cell adhesion molecule-1 and some selectins, thus inhibiting the interaction of endothelial cells and neutrophils [10, 11].

In this study, PGE1 pretreatment reduced the expression of the proapoptotic protein Bax and increased the expression of antiapoptotic protein Bcl-2 after reperfusion, which hinted PGE1 could reduce the apoptosis of liver cells. Bax-dependent mitochondrial outer membrane permeabilization is regulated by the interaction between proapoptotic and antiapoptotic members of Bcl-2 family [28]. After Bax is activated, it combines with the mitochondrial outer membrane and further prompts mitochondria to release caspase 3, cytochrome c, and other apoptosis inducing factors [29]. Bax also can form dimers with Bcl-2, which prompts the release of Ca^2+^ from the endoplasmic reticulum, and interacts with cytochrome c to activate caspase signaling pathways that can produce apoptotic bodies and induce apoptosis [30]. Bcl-2, the terminal part of apoptotic regulation, can adjust the redox state of cells to prevent oxidative damage of cell components and DNA rupture. It can also inhibit the transmembrane transport of Ca^2+^, the release of cytochrome c from mitochondria, and other factor-mediated apoptosis [31].

## 5. Conclusion

In this study, we found that PGE1 preconditioning protects liver IR injury with extrahepatic cholestasis by improving hepatic microcirculation and reducing intrahepatic neutrophil infiltration, oxidative damage, and hepatocyte apoptosis. This study still has some limitations. For example, we have not done researches at the genetic levels and signaling pathways and also have not evaluated the liver function such as bile production after 24 h reperfusion. For the goal to apply in a clinical condition, we also need to perform further research using a large animal model.
